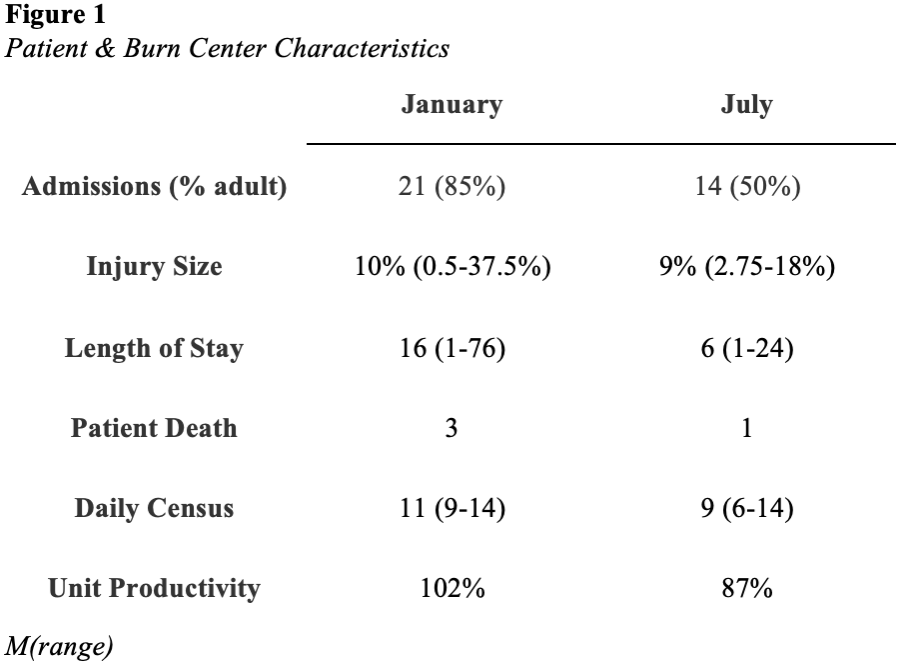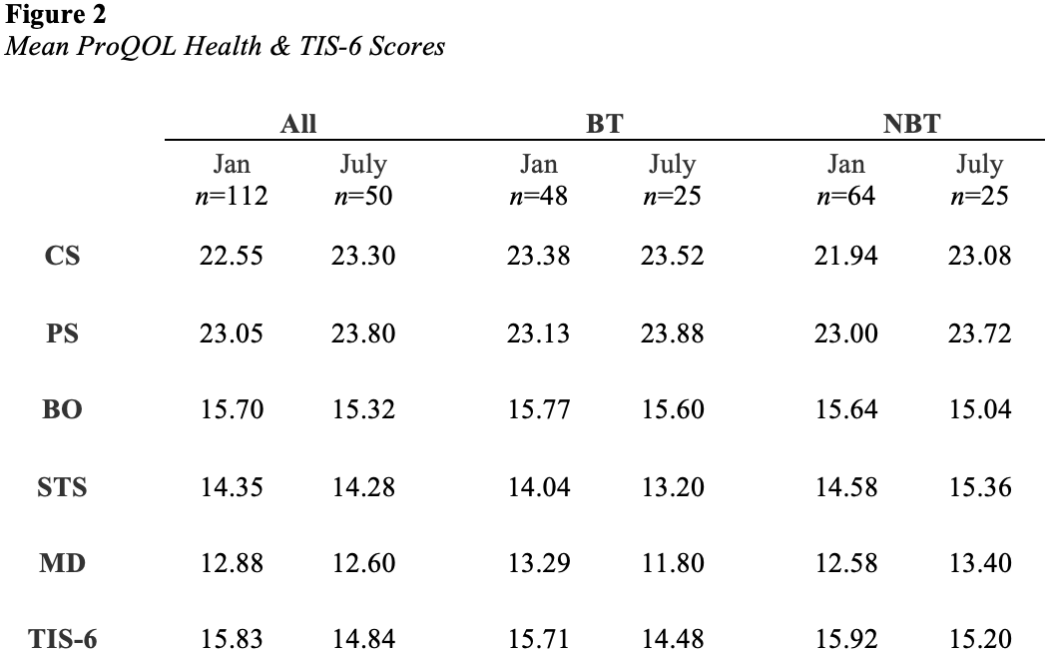# 916 Impact of Patient and Center Factors on Providers’ Professional Quality of Life and Turnover Intention

**DOI:** 10.1093/jbcr/iraf019.447

**Published:** 2025-04-01

**Authors:** Christine Grauer, Miranda Yelvington, Beverly Spray

**Affiliations:** Arkansas Children’s Hospital; Arkansas Children’s Hospital; Arkansas Children’s Hospital

## Abstract

**Introduction:**

Healthcare providers’ professional quality of life (ProQOL) may reflect individuals’ personalities, patient characteristics, and environmental factors. This study aimed to compare the impact of patient- and care-related factors on healthcare providers’ ProQOL and intent to leave between periods of high and low census in one calendar year at a single Burn Center (BC).

**Methods:**

Multidisciplinary healthcare providers who care for adult and pediatric burn survivors completed a demographic, Professional Quality of Life Health Measure (ProQOL Health), and Turnover Intention Scale (TIS-6) survey in January and July 2024, periods of high and low census respectively. Aggregate patient and BC data for the corresponding survey periods were summarized. The ProQOL Health includes five subscales: Compassion Satisfaction (CS), Perceived Support (PS), Burnout (BO), Secondary Traumatic Stress (STS), and Moral Distress (MD). Subscale scores are defined as low ≤ 12, moderate 13-23, and high ≥ 24. TIS-6 scores ≥ 18 represent an intent to leave.

**Results:**

Patient- and care-related differences between January and July 2024 were summarized (Figure 1). Mean TIS-6 and ProQOL Health subscale scores were calculated (Figure 2). Further statistical analysis between participants who identified as Burn Team (BT) and Non-Burn Team (NBT) members will be available at abstract presentation.

Figure 1 Patient & Burn Center Characteristics

Figure 2 Mean ProQOL Health & TIS-6 Scores

This repeated cross-sectional study demonstrated moderate levels of CS, PS, BO, STS, and MD and low turnover intention among healthcare providers who care for adult and pediatric burn survivors. Mean BT MD scores were higher during high census (13.29) compared to scores during low census (11.80). Mean BT CS scores (23.38) were higher than mean NBT CS scores (21.94) when census was high.

**Conclusions:**

Higher MD scores among BT members may be related to patient deaths or care of patients with larger injuries and longer hospitalizations. Higher CS scores among BT members may reflect the enjoyment these staff members derive from caring for others and feeling successful in their work.

**Applicability of Research to Practice:**

Understanding the impact of factors when BC census changes can inform leaders’ choices of targeted interventions to promote staff compassion satisfaction and reduce feelings of moral distress.

**Funding for the Study:**

Organization-Specific Research Grant